# Supplementary material for: Intracellular Fusobacterium nucleatum infection attenuates antitumor immunity in esophageal squamous cell carcinoma
Source: Nat Commun. 2023 Sep 18;14:5788. doi: 10.1038/s41467-023-40987-3 (PMC10507087; doi:10.1038/s41467-023-40987-3)
Supplement: Supplementary file 1 — Supplementary Information [file 41467_2023_40987_MOESM1_ESM.pdf]

Supplemental Information

Intracellular *Fusobacterium nucleatum* infection attenuates antitumor immunity in esophageal squamous cell carcinoma

Yiqiu Li[1], Shan Xing[2], Fangfang Chen[1], Qifan Li[1], Shuheng Dou[1], Yuying Huang[1], Jun An[3*], Wanli Liu[2*], Ge Zhang[1*]

[1]Department of Microbial and Biochemical Pharmacy, School of Pharmaceutical Sciences, Sun Yat-sen University, Guangzhou, China.

[2]Department of Clinical Laboratory, State Key Laboratory of Oncology in South China, Collaborative Innovation Center for Cancer Medicine, Sun Yat-sen University Cancer Center, Guangzhou, China.

[3]Department of Cardiothoracic Surgery, The Third Affiliated Hospital of Sun Yat-sen University, Yuedong Hospital, Guangzhou, China.

Fig.S1 Representative images of immunohistochemistry staining of PD-L1 expression in the NR group

Fig.S2 Representative images of immunohistochemistry staining of PD-L1 expression in the R group

Fig.S3 Immunohistochemistry positive rates of PD-L1 in ESCC paraffin sections

Fig.S4 *F. nucleatum* promotes AKR cell metastasis and decreases the efficacy of αPD-L1 *in vivo*

Fig.S5 *F. nucleatum* infection decreases the effectiveness of αPD-L1 in female tumor-bearing mice

Fig.S6 *F. nucleatum* infection decreases the effectiveness of αPD-L1 in larger tumor-bearing mice

Fig.S7 *F. nucleatum* infection attenuated T-cell activation *in vivo*

Fig.S8 *F. nucleatum* protects ESCC cells against direct cytotoxicity from splenocytes *in vitro*

Fig.S9 The effect of *F. nucleatum* infection on PBMCs, human $CD8^+$ T cells and Jurkat cells

Fig.S10 Human $CD8^+$ T cells were obtained from PBMCs

Fig.S11 The effect of lipopolysaccharides (LPS) in Jurkat cells

Fig.S12 Only live *F. nucleatum* could survive and upregulate the expression of PD-L1 in ESCC cells

Fig.S13 Dichromatic IF staining of PD-L1 in ESCC cells

Fig.S14 PD-L1 transcription factor prediction, ATF3 gene exploration from TCGA database, ATF3 siRNA efficiency validation and ATF3-binding event prediction

Fig.S15 IF staining of PD-L1 or ATF3 in tumor tissues from C57BL/6 xenografts

Fig.S16 Validation of Fn and Fn-Dps antibodies specificity

Fig. S17 Validation of Fn primer specificity

Table S1: ESCC patients details for ELISA

Table S2: ESCC patients details for PD-L1 and Fn detection

Table S3: All antibodies used in flow cytometry analysis

Table S4: All antibodies used in histology and immunofluorescence

Table S5: The sequences of primers for the qPCR analysis

Table S6: All antibodies used in western blotting and Co-IP

Table S7: Table summary of differentially expressed genes, related to Figure 6E

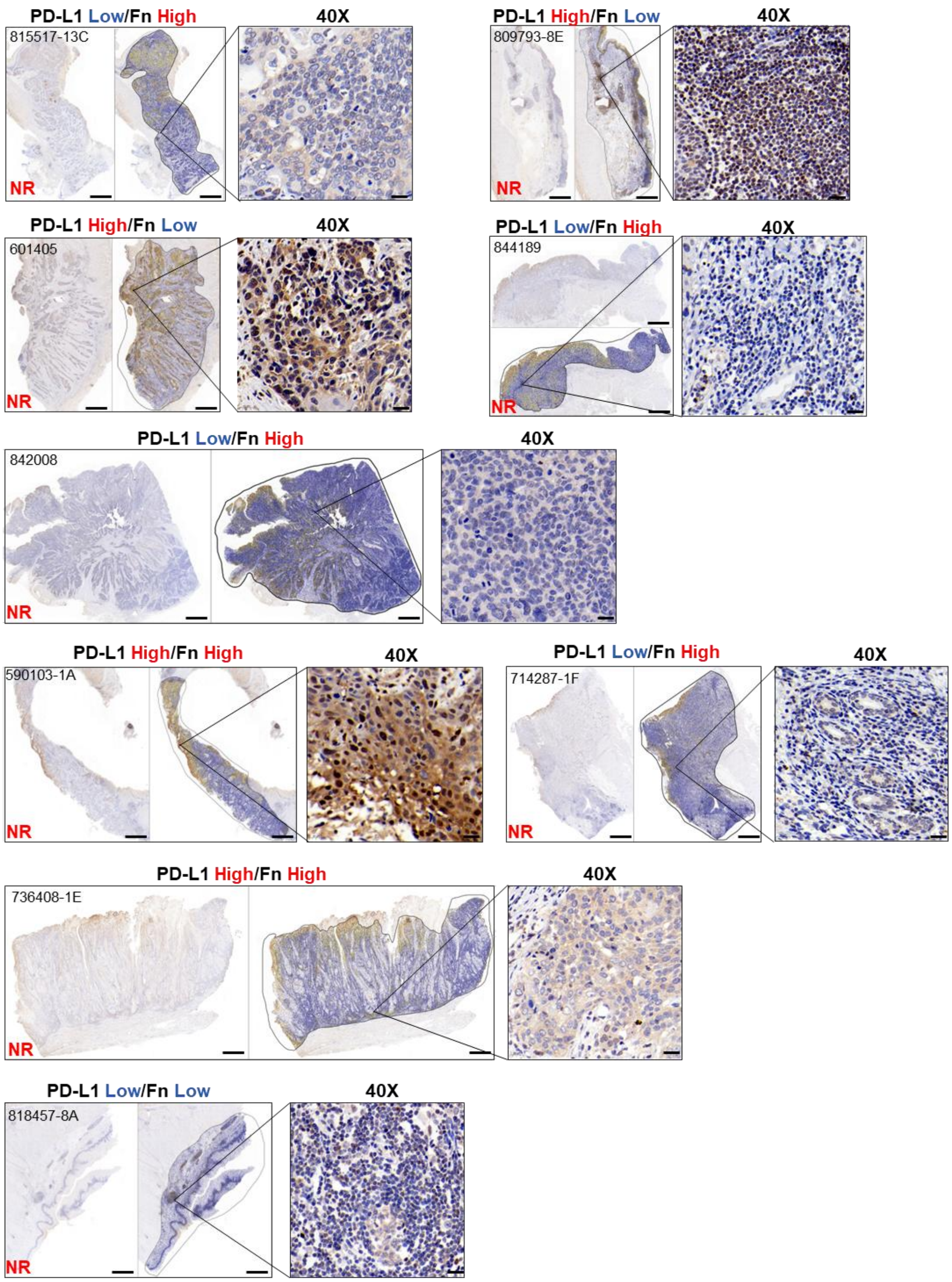

Figure S1 Representative images of immunohistochemistry staining of PD-L1 expression in the NR group

Scale bar: 2000 μm and 20 μm (40×). Images were representative results of n = 2 independent experiments with similar results.

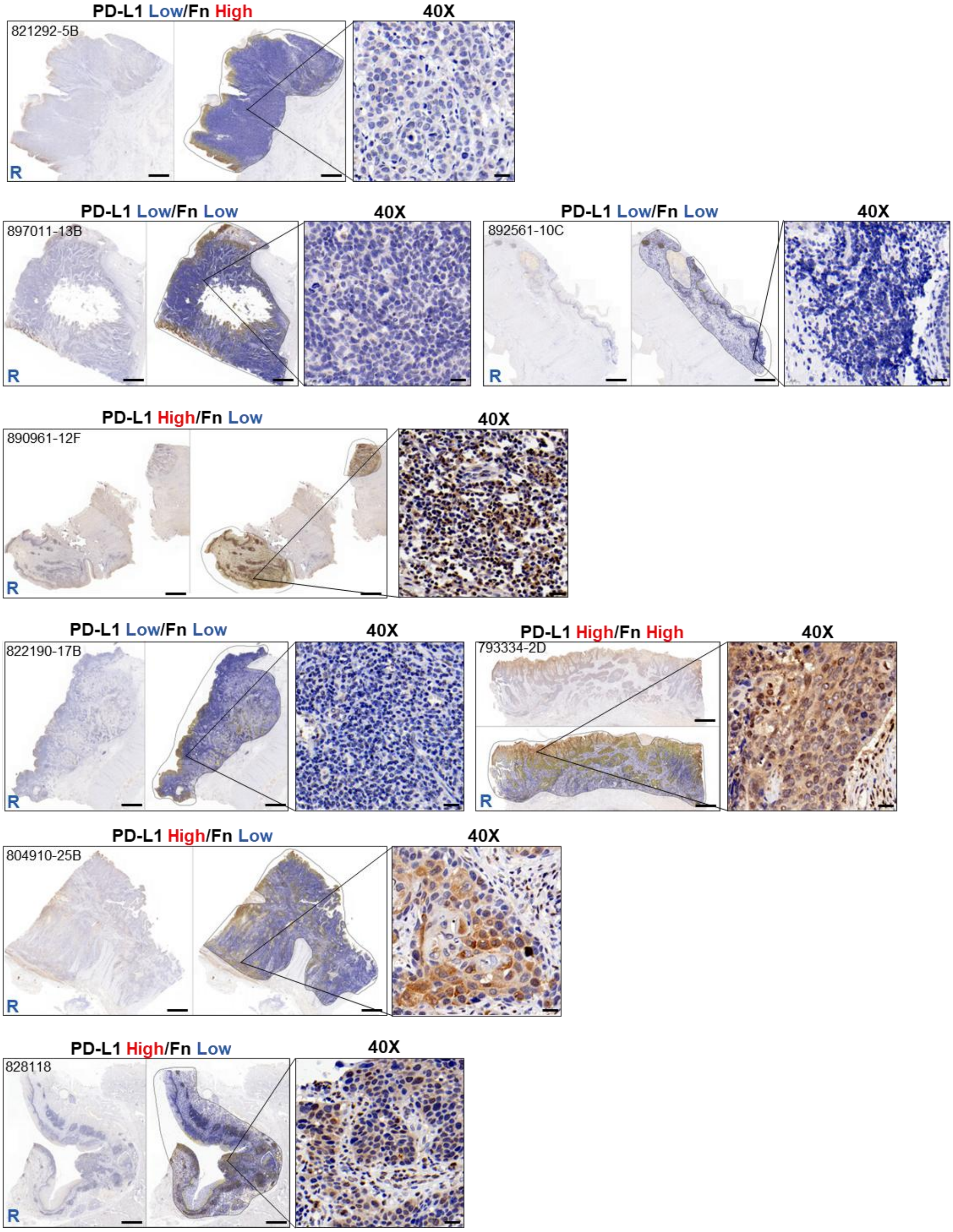

Figure S2 Representative images of immunohistochemistry staining of PD-L1 expression in the R group

Scale bar: 2000 μm and 20 μm (40×). Images were representative results of n = 2 independent experiments with similar results.

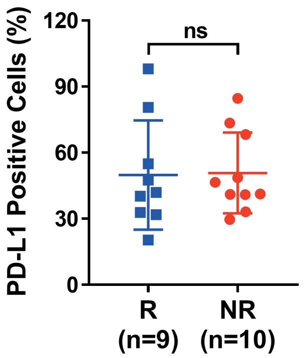

Figure S3 Immunohistochemistry positive rates of PD-L1 in ESCC paraffin sections

The statistical significance of result was determined by a two-tailed unpaired Mann-Whitney test.

(Mean ± SD; n = 9 in R group, n = 10 in NR group)

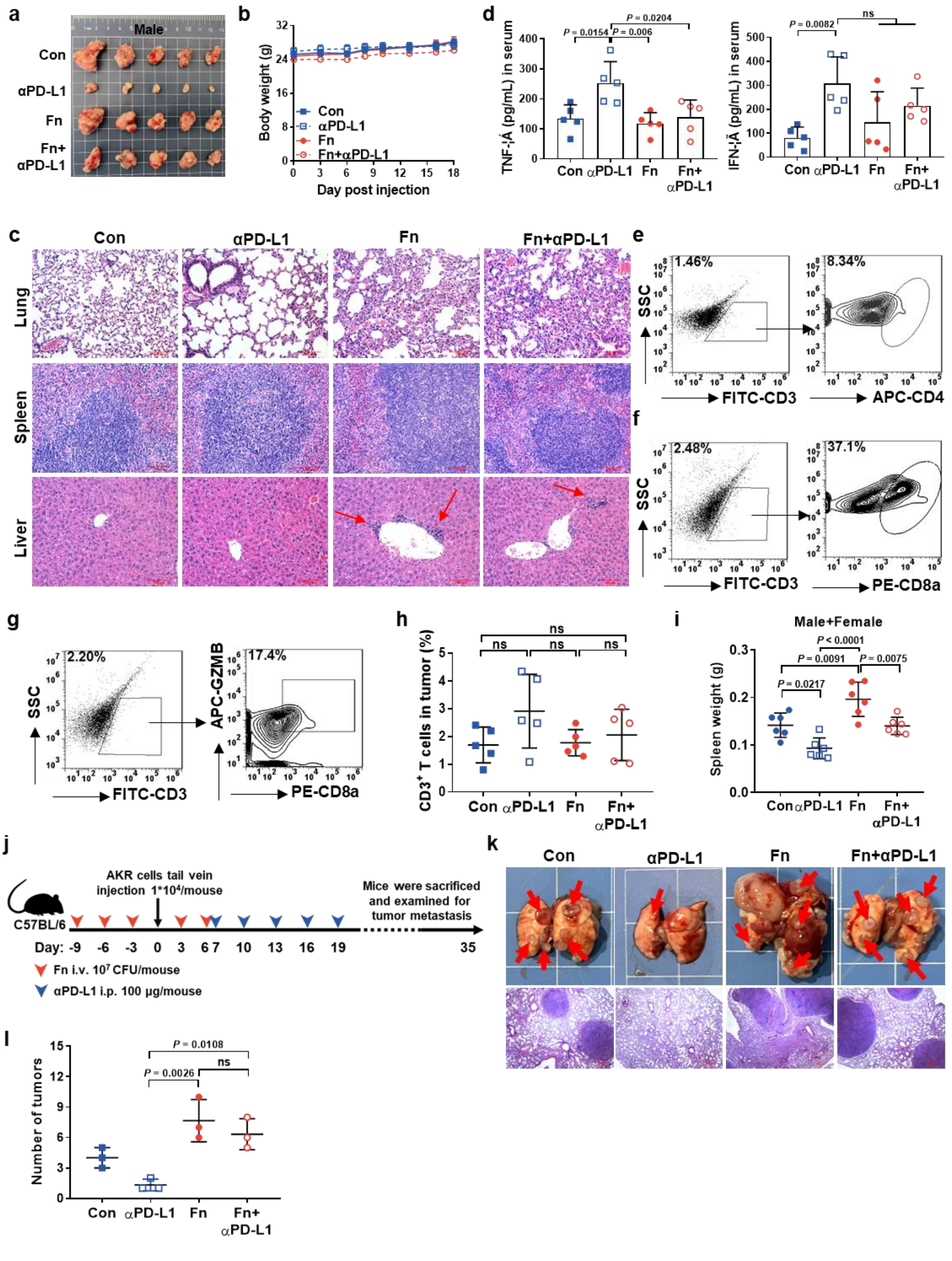

Figure S4 ***F. nucleatum*** **promotes AKR cell metastasis and decreases the efficacy of αPD-L1** ***in vivo***

(a-d) C57BL/6 mice (5 male per group) were implanted with 5 × 10^6 AKR cells after Fn infection (*i.v.*, 10^7 CFU/mouse) three times. αPD-L1 (*i.p.*, 100 μg/mouse) once every three days.

Representative tumor images (a). Mean body weight ± SEM. (b). H&E staining analysis in lung, spleen and liver from C57BL/6 xenografts. Scale bar: 100 μm (c). Measurement of TNF-α and IFN-γ in mouse serum by ELISA (n = 5 per group) (d). ns means not significant.

(e-h) FACS of $CD4^+$ cells in $CD3^+$ cells (e), $CD8a^+$ cells in $CD3^+$ cells (f), $GZMB^+CD8a^+$ cells in $CD3^+$ TILs (g) in tumors and quantification of $CD3^+$ T cells in tumors (h; mean ± SD; n = 1 experiment; n = 5 mice in each group). ns means not significant.

(i) Summary of spleen weight data of C57BL/6 xenografts (the experiment was done once; n = 6 mice in each group; n = 1 experiment; n = 6 mice in each group).

(j-l) C57BL/6 mice (n = 1 experiment; n = 3 mice in each group) were tail vein injected with 5×10^4 AKR cells. Fn infection (*i.v.*, 10^7 CFU/mouse) five times. αPD-L1 (*i.p.*, 100 μg/mouse) five times. A schematic view of the administration plan (j). Representative images and H&E staining of lung metastases (scale bar = 100 μm) (k) and quantification (l, mean ± SD). ns means not significant.

The statistical significance of results in figure d, h, i and l was determined by a one-way ANOVA analysis.

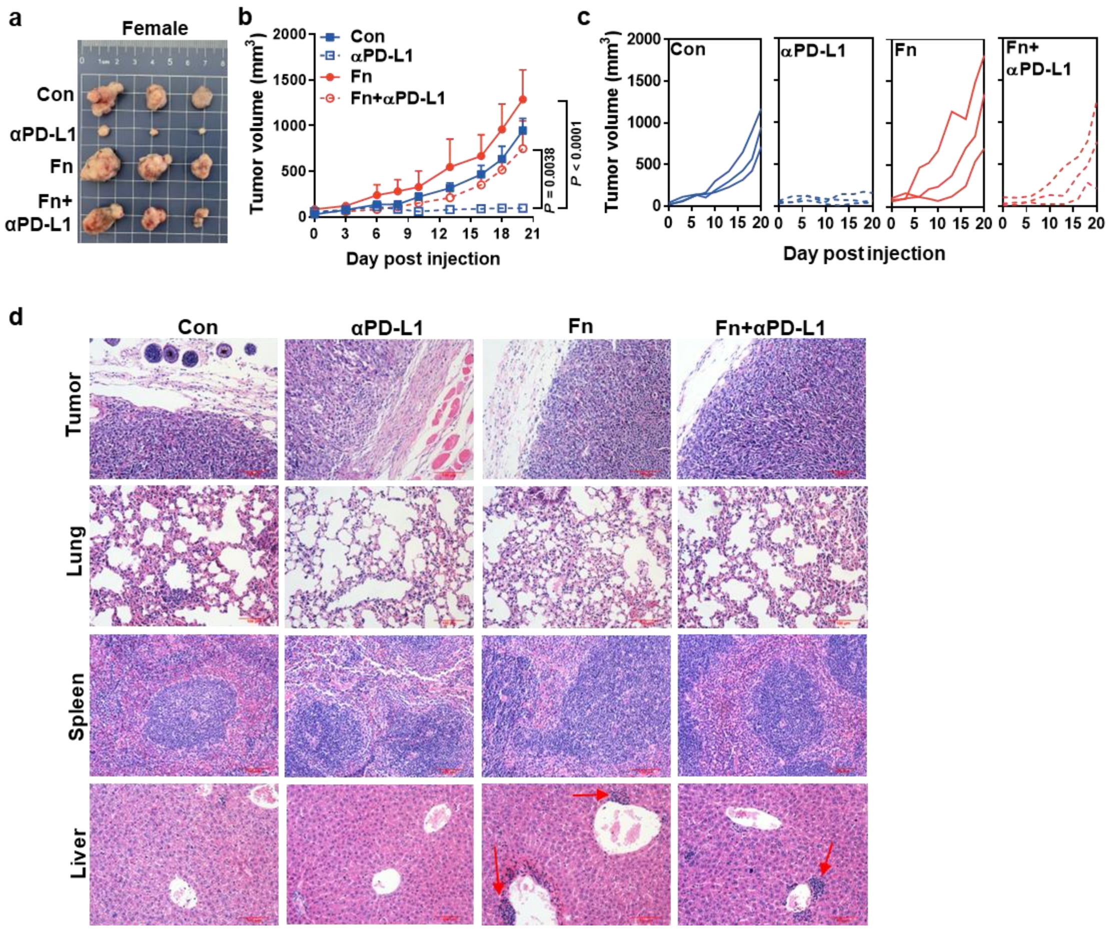

Figure S5 *F. nucleatum* infection decreases the effectiveness of αPD-L1 in female tumor-bearing mice

(a-d) C57BL/6 mice (the experiment was done once; n = 3 mice in each group) were implanted with 5 × 10^6 AKR cells after Fn infection (*i.v.*, 10^7 CFU/mouse) three times. αPD-L1 (*i.p.*, 100 µg/mouse) once every three days. Representative tumor images (a). The mean tumor volume ± SEM (b). Each tumor volume (c). H&E staining analysis in tumor, lung, spleen and liver from C57BL/6 xenografts. Scale bar: 100 µm (d).

Statistical significance in figure b was determined by two-way ANOVA analysis for comparison at the endpoint.

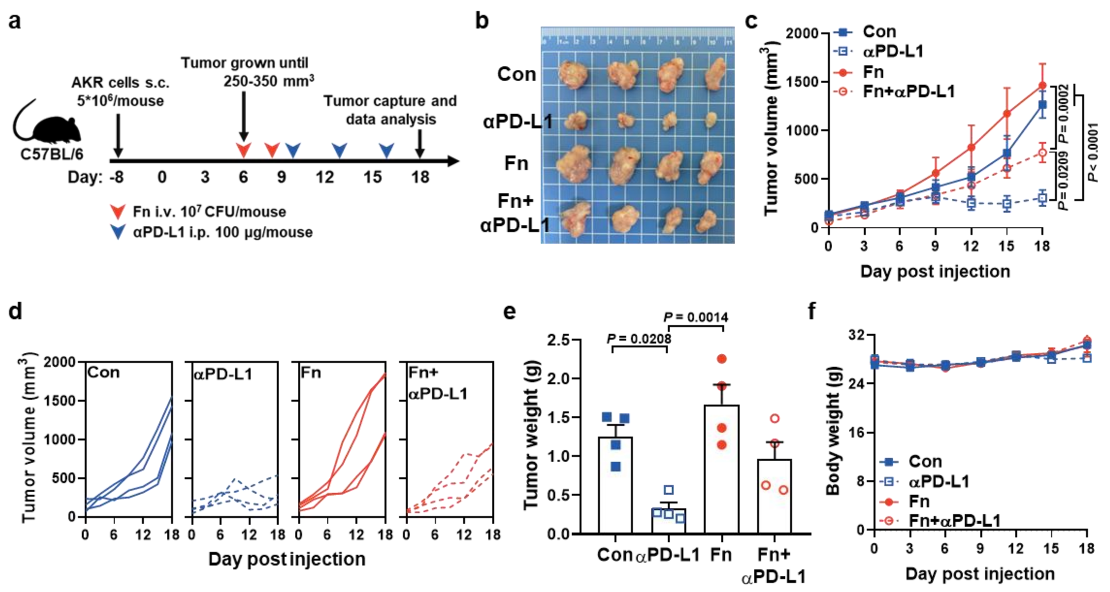

Figure S6 ***F. nucleatum*** **infection decreases the effectiveness of αPD-L1 in larger tumor-bearing mice**

(a-f) C57BL/6 mice (the experiment was done once; n = 4 mice in each group) were implanted with 5 × 10^6 AKR cells before Fn infection (*i.v.*, 10^7 CFU/mouse) twice. αPD-L1 (*i.p.*, 100 µg/mouse) once every three days. A schematic view of the administration plan (a). Representative tumor images (b). The mean tumor volume ± SEM (c). Each tumor volume (d). Mean tumor weight ± SEM (e). Mean body weight ± SEM (f).

Statistical significance in figure c was determined by two-way ANOVA analysis for comparison at the endpoint. Statistical significance in figure e was determined by a one-way ANOVA analysis.

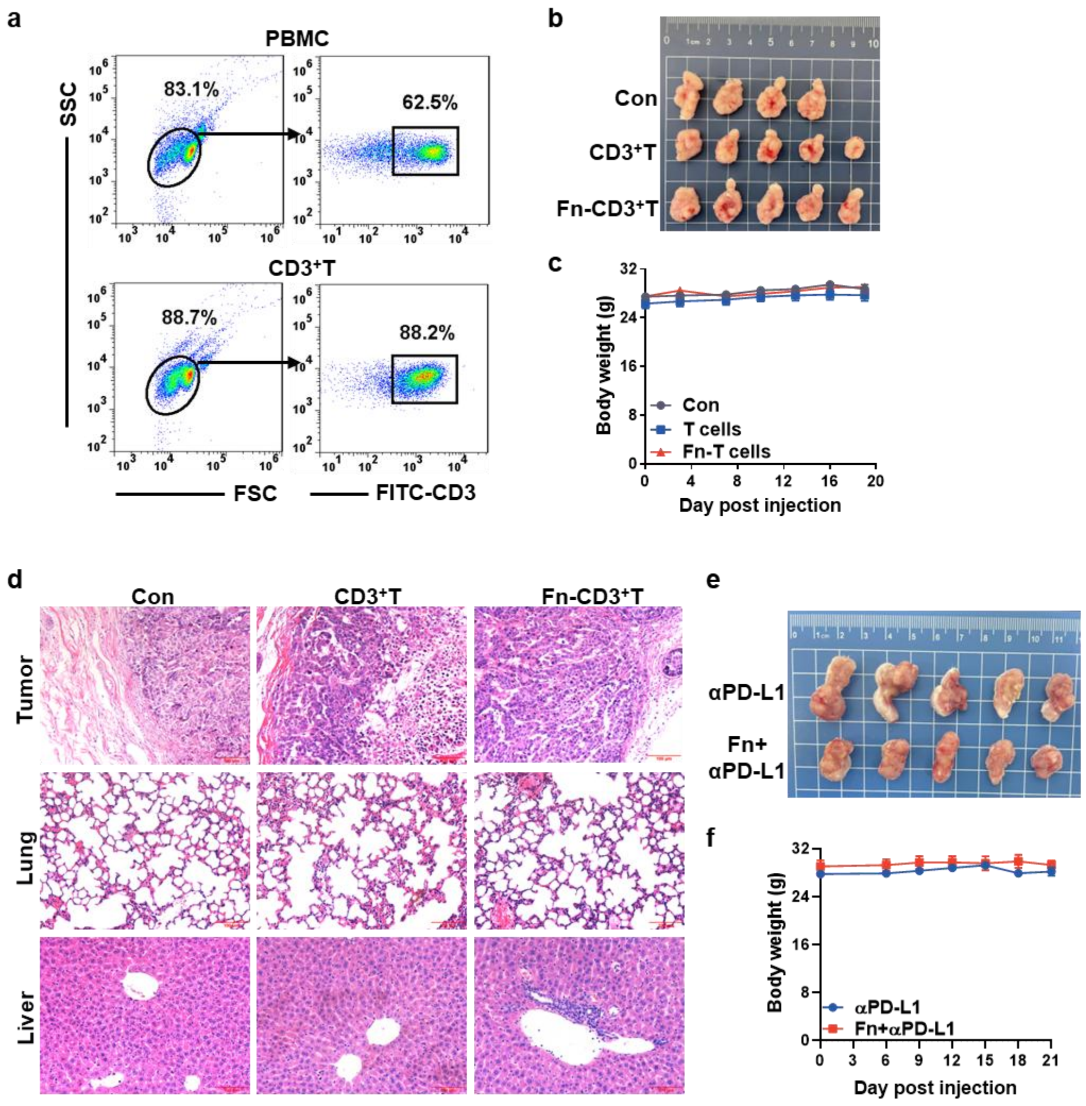

Figure S7 ***F. nucleatum*** **infection attenuated T-cell activation** ***in vivo***

(a) Human $CD3^+$ T cells were obtained and purified from human peripheral blood mononuclear cells (PBMCs) using CD3 microbeads according to the manufacturer's instructions.

(b-d) NSG mice (the experiment was done once; n = 4 mice in Con group and n = 5 mice in other groups) were implanted with 5×10^6 E109 cells, and then the mice were tail vein injected with 2×10^6 human $CD3^+$ T cells or Fn-$CD3^+$ T cells (Fn preinfection for 24 h) when the average tumor size reached 250 mm^3. Representative tumor images (b). Mean body weight ± SEM (c). H&E staining analysis of tumors, lungs and livers from NSG mice. Scale bar: 100 μm (d).

(e-f) NSG mice (the experiment was done once; n = 5 mice in each group) were implanted with 5 × 10^6 E109 cells and Fn infection (*i.v.*, 10^7 CFU/mouse) three times. Then the mice were tail vein injected with 2 × 10^6 human $CD3^+$ T cells. αPD-L1 (*i.p.*, 100 µg/mouse) once every three days. Representative tumor images (e). Mean body weight ± SEM (f).

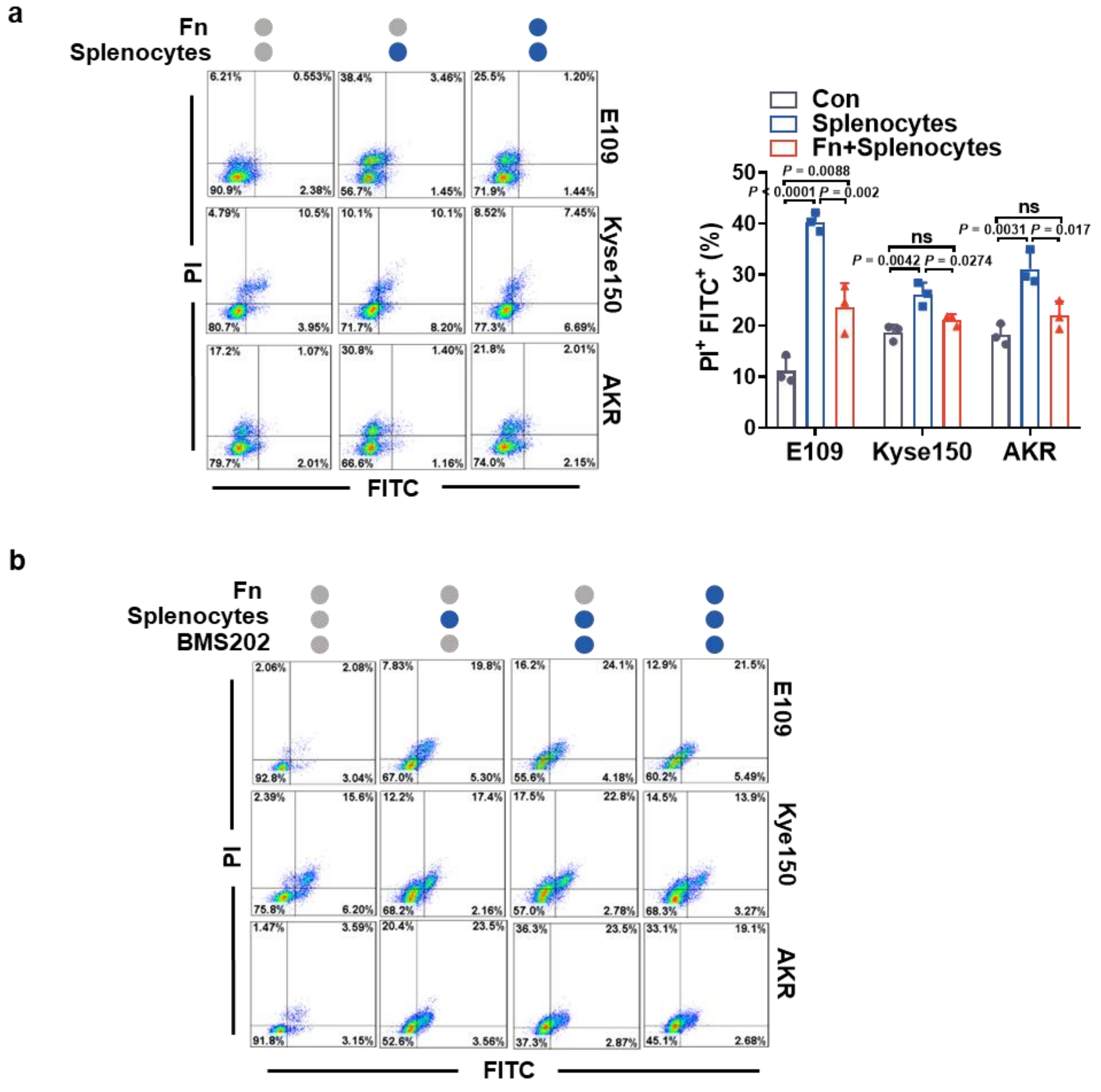

Figure S8 *F. nucleatum* protects ESCC cells against direct cytotoxicity from splenocytes *in vitro*

(a-b) ESCC cells were pre-infected with Fn for 48 h before coculture with splenocytes for 60 h (mean ± SD; n = 3 biological replicates). Cells were pretreated with BMS202 (1 mM), a PD-1/PD-L1 blockade, 2 h before splenocytes were added. Labeling patterns indicated different cell populations, with FITC- and PI-negative cells representing viable tumor cells, the other quadrants representing apoptotic and necrotic tumor cells. ns means not significant.

Statistical significance in figure a was determined by a one-way ANOVA analysis.

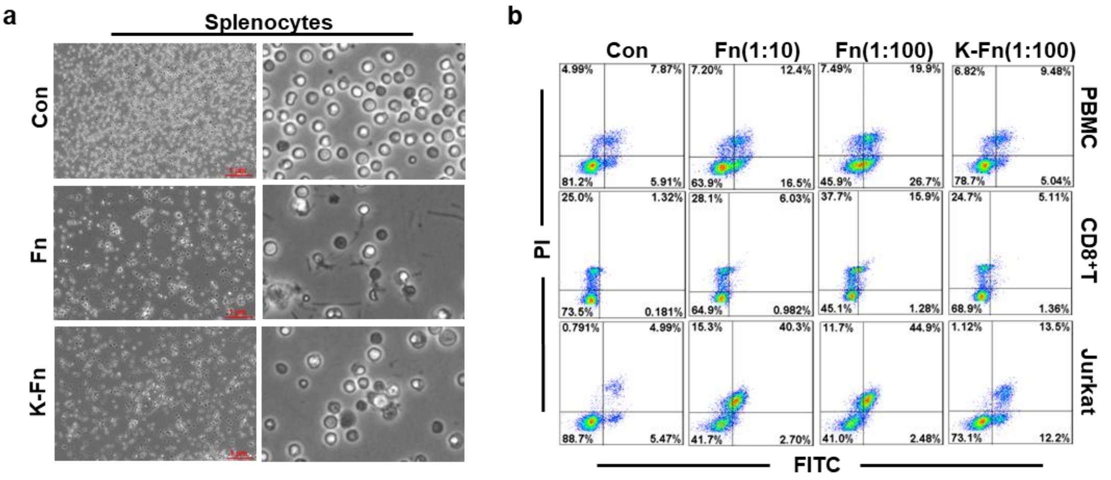

Figure S9 The effect of *F. nucleatum* infection on PBMCs, human CD8$^+$ T cells and Jurkat cells

(a) Representative images of splenocytes infected with Fn or heat-killed Fn for 48 h (n = 3 independent experiments with similar results). Scale bar: 1 μM (left).

(b) Annexin V-FITC/PI-positive apoptotic cells treated with Fn (MOI of 1:10 or 1:100) or heat-killed Fn (MOI of 1:100) were counted by flow cytometry with Annexin V-FITC/PI double staining.

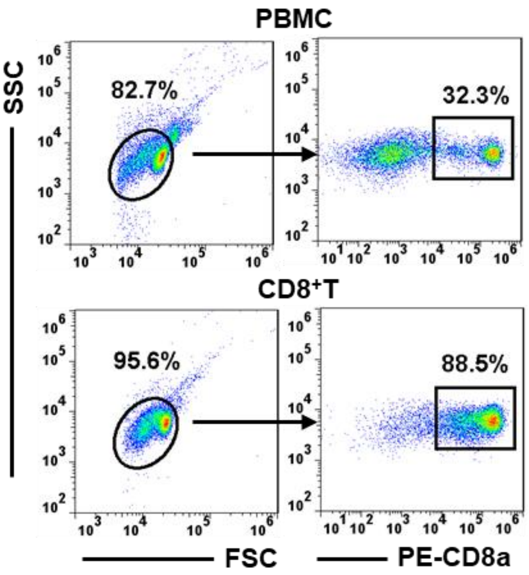

Figure S10 Human CD8$^+$ T cells were obtained from PBMCs

Human CD3$^+$ T cells were obtained and purified from human peripheral blood mononuclear cells (PBMCs) using CD3 microbeads according to the manufacturer's instructions.

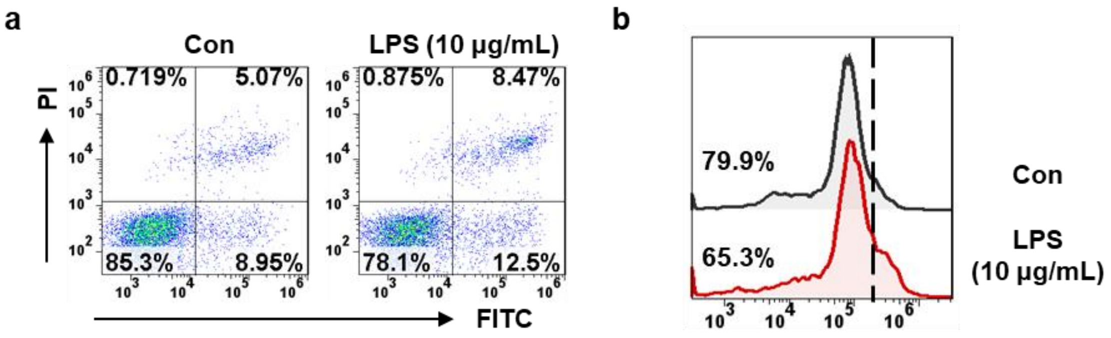

Figure S11 The effect of lipopolysaccharide (LPS) in Jurkat cells

(a) Annexin V-FITC/PI-positive apoptotic cells treated with LPS (10 μg/mL, Sigma–Aldrich, MO, USA) for 48 h were counted by flow cytometry with Annexin V-FITC/PI double staining (n = 3 biological replicates).

(b) Proliferation of Jurkat cells was analyzed by cell trace CFSE (CTC) dilution after treatment with LPS (10 μg/mL) for 48 h (n = 3 biological replicates).

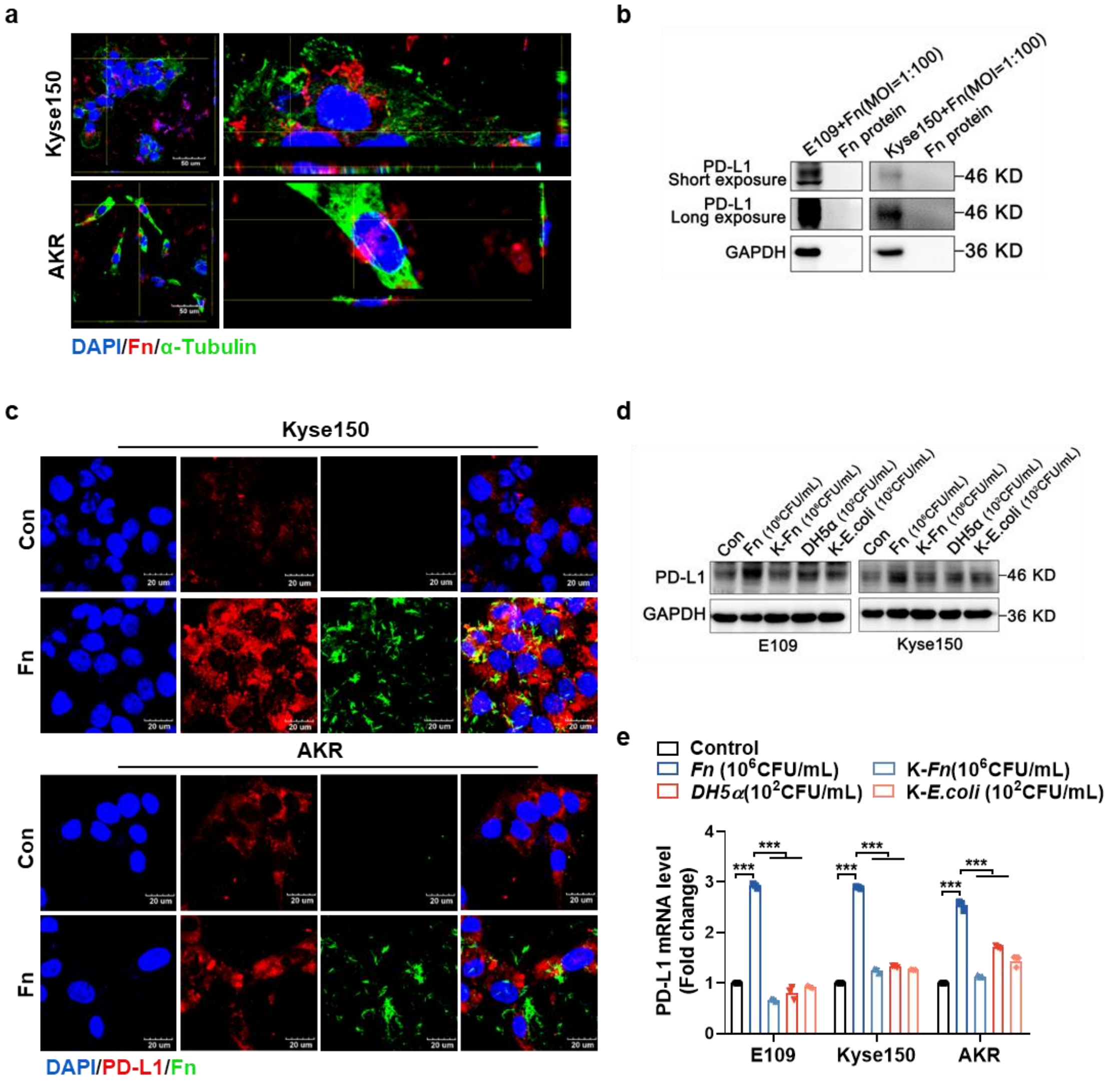

Figure S12 Only live *F. nucleatum* could survive and upregulate the expression of PD-L1 in ESCC cells

(a) Three-dimensional visualization of α-tubulin and Fn in Kyse150 and AKR cells . Cells were infected with Fn (MOI of 1:10) for 48 h. Scale bar: 50 μm.

(b) Immunoblotting analysis of PD-L1 in E109 and Kyse150 cells and whole Fn protein. Cells were infected with Fn (MOI of 1:100) for 48 h.

(c) Dichromatic IF staining of PD-L1 and Fn in Kyse150 and AKR cells. Cells were infected with Fn (MOI of 1:10) for 48 h. Scale bar: 20 μm.

(d-e) Immunoblotting and qRT–PCR analysis of PD-L1 in E109 and Kyse150 cells. Cells were

infected with Fn, heat-killed Fn*, DH5α or* heat-killed *E. coli* for 48 h. Results are presented as mean ± SD of three independent experiments, *** $P < 0.0001$.

Images in a-c were representative results of n = 3 independent experiments with similar results. The statistical significance of result in figure e was determined by two-way ANOVA multiple comparisons.

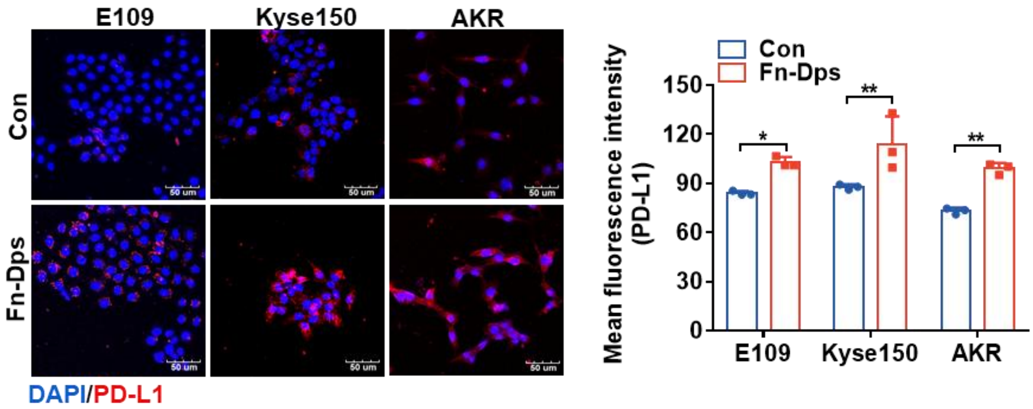

Figure 13 Dichromatic IF staining of PD-L1 in ESCC cells

Cells were treated with Fn-Dps (1 μM) for 48 h (mean ± SD; n = 3 biological replicates). Scale bar: 50 μm. The statistical significance of result was determined by a two-tailed unpaired t test.

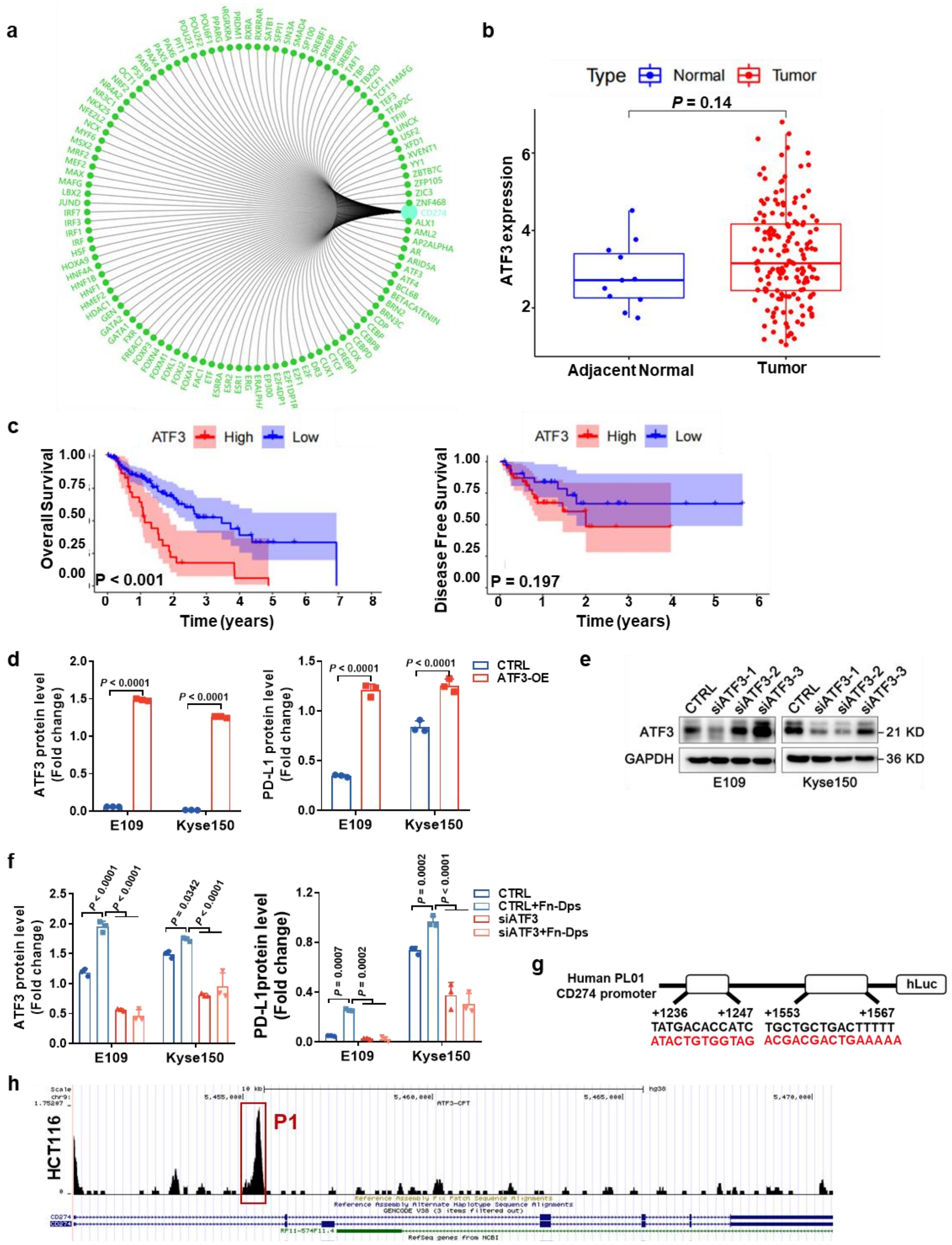

Figure S14 PD-L1 transcription factor prediction, ATF3 gene exploration from TCGA database, ATF3 siRNA efficiency validation and ATF3-binding event prediction

(a) PD-L1 transcription factor prediction from www.gcbi.com.cn.

(b) Differential expression of ATF3 between tumor tissues (n = 162) and adjacent normal tissues

(n = 11), adjacent normal tissue is represented in blue, whereas tumor tissue is shown in red. Box and whisker plot; boxes depict the upper and lower quartiles of the data, and whiskers depict the range of the data.

(c) OS and DFS analysis of ATF3 in the high and low expression groups (mean ± SEM).

(d) The quantification of Fig 5H (mean ± SD; n = 3 biological replicates). ESCC cell lines (E109 and Kyse150) were transfected with ATF3 overexpression (OE) or negative control (CTRL) vectors, and ATF3 and PD-L1 expression was analyzed by western blotting.

(e) Immunoblotting analysis of ATF3 in E109 and Kyse150 cells transfected with siRNA against ATF3 for 48 h (mean ± SD; n = 3 biological replicates).

(f) The quantification of Fig 5J (mean ± SD; n = 3 biological replicates). Immunoblotting analysis of ATF3 and PD-L1 in E109 and Kyse150 cells. Cells were treated with Fn-Dps (1 μM) for 48 h after transfection with siRNA against ATF3 for 48 h.

(g) Schematic representation of the *CD274* promoter cloned into the pGL3 vector. Two predicted ATF3 binding motifs are shown, and promoter constructs containing mutations in these two regions to cause ATF3-binding deficiency are generated.

(h) Genome browser display of ATF3-binding events on the promoter and body of CD274 (PD-L1) in HCT116 cells, data from Zhao J, et al. BMC Genomics 2016.

Statistical significance in figure c was determined by Kaplan-Meier analysis. The statistical significance of results in figure d was determined by a two-tailed unpaired t test. f was determined by one-way ANOVA analysis.

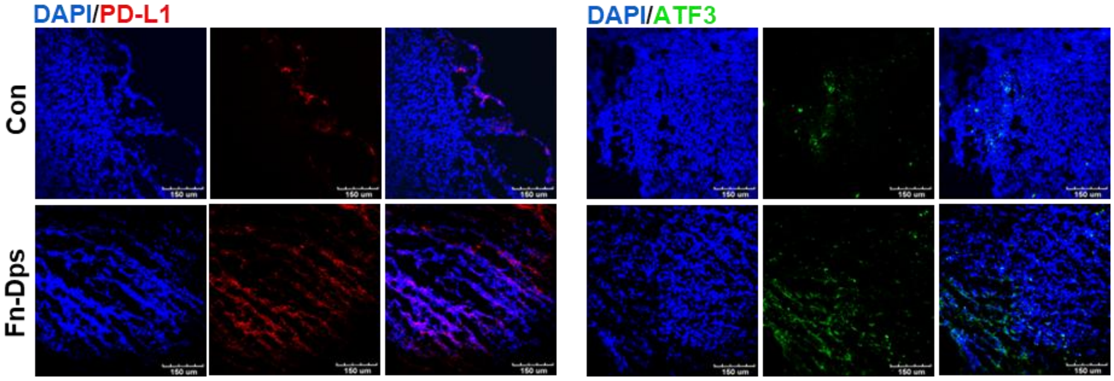

Figure S15 IF staining of PD-L1 or ATF3 in tumor tissues from C57BL/6 xenografts.

Scale bar: 150 μm. Images were representative results of n = 3 independent experiments with similar results.

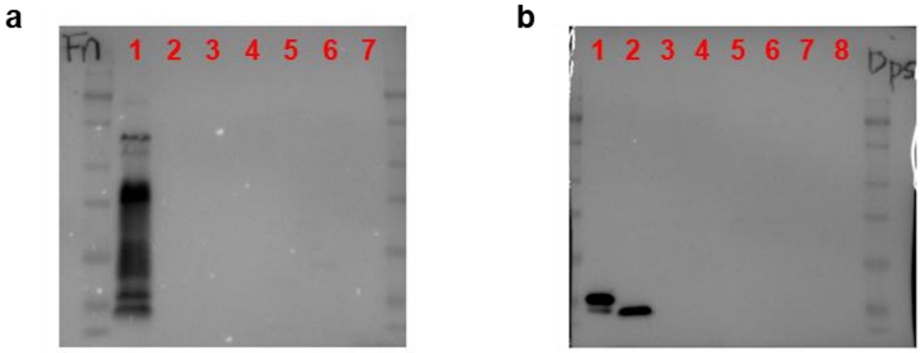

Figure S16 Validation of Fn and Fn-Dps antibodies specificity.

The specificity of Fn and Fn-Dps antibodies were confirmed by using immunoblotting experiments, equal protein loading for all bacterial samples.

(a) The specificity of Rabbit anti-Fn (dilution ratio: 1:50000). 1, *Fusobacterium nucleatum*; 2, *Clostridium ventriculi*; 3, *Bifidobacterium bifidum*; 4, *Bifidobacterium longum*; 5, *Parabacteroides distasonis*; 6, *Akkermansia muciniphila*; 7, *Porphyromonas gingivalis*.

(b) The specificity of Mouse anti-Fn-Dps (dilution ratio: 1:20000). 1, Fn-Dps 2, *Fusobacterium nucleatum*; 3, *Clostridium ventriculi*; 4, *Bifidobacterium bifidum*; 5, *Bifidobacterium longum*; 6, *Parabacteroides distasonis*; 7, *Akkermansia muciniphila*; 8, *Porphyromonas gingivalis*.

Images in a and b were representative results of n = 3 independent experiments with similar results.

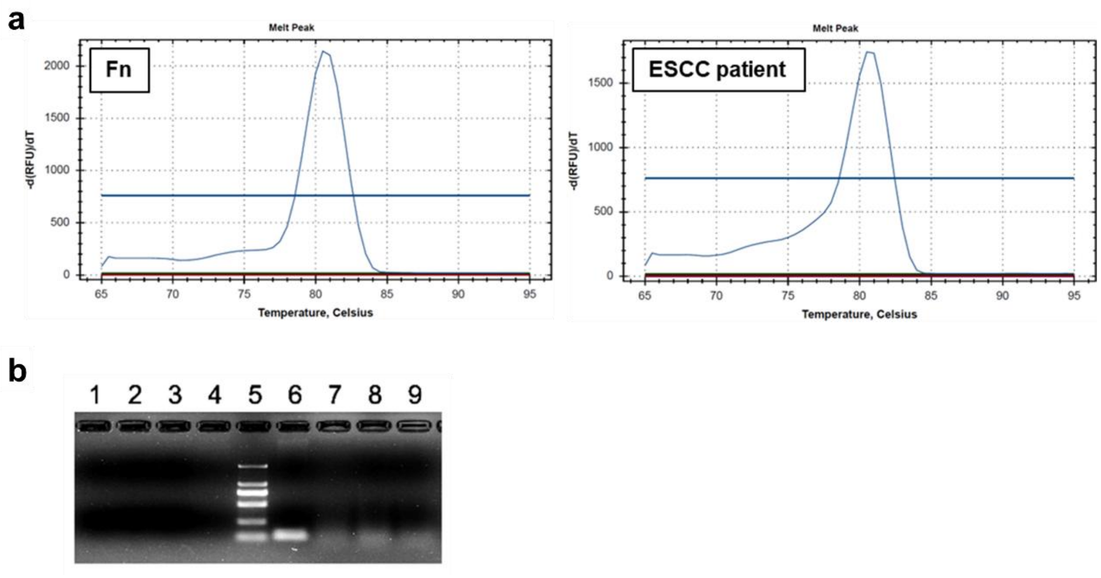

Figure S17 Validation of Fn primer specificity.

(a) qRT-PCR specificity of Fn primer was verified by melting curve analysis. ESCC patient (ID: 844189)

(b) PCR amplification was visually confirmed using 2 % agarose gel. 1, *Porphyromonas gingivalis*; 2, *Bifidobacterium bifidum*; 3, *Akkermansia muciniphila*; 4, Negative control; 5, DL2000; 6, *Fusobacterium nucleatum*; 7, ESCC patient (ID: 601405); 8, ESCC patient (ID: 821292-5B); 9, ESCC patient (ID: 844189).

Images in a and b were representative results of n = 3 independent experiments with similar results.

Table S1: ESCC patients details for ELISA

ID	Age	Male(M) or Female(F)	Curative Effect	Treatment	First αPD-1 treatment time	Fn-IgG$_{(OD450nm)}$
PD252	61	M	PR	αPD-1	19/9/2020	0.055
PD674	42	M	PR	αPD-1	24/12/2020	0.103
PD245	63	M	PR	PTX+S-1+αPD-1	2/6/2020	0.250
PD853	63	M	PR	αPD-1	6/1/2021	0.120
PD865	69	M	PR	αPD-1	23/1/2021	0.332
PD1255	51	F	PR	αPD-1	3/4/2021	0.504
E4	60	M	PR	PTX+Capecitabine+αPD-1	28/11/2020	0.126
E5	71	F	PR	αPD-1+IP	4/12/2020	0.146
E8	61	F	PR	αPD-1	10/3/2021	0.177
E10	61	M	PR	PTX+Capecitabine+αPD-1	16/3/2021	0.189
E22	60	M	PR	αPD-L1+EP	13/4/2021	0.548
E27	63	F	PR	PTX+Nedaplatin+αPD-1	22/4/2021	0.357
E30	57	F	PR	EGFR+PTX+Cisplatin	28/4/2021	0.279
E31	57	M	PR	PTX+Nedaplatin+αPD-1	29/4/2021	0.149
E37	72	M	PR	PTX+Nedaplatin+αPD-1	18/5/2021	0.572
E38	73	M	PR	PTX+Nedaplatin+αPD-1	18/5/2021	0.279
E41	51	M	PR	PTX+Cisplatin+αPD-1	27/5/2021	0.115
E45	72	M	PR	PTX+Nedaplatin+αPD-1	31/5/2021	0.523
E48	62	M	PR	PTX+Cisplatin+αPD-1	22/6/2021	0.303
E49	72	M	PR	Capecitabine+αPD-1	23/6/2021	0.208
E65	66	M	PR	PTX+Nedaplatin+αPD-1	29/7/2021	0.384
E70	64	M	PR	SOX+αPD-1	5/8/2021	0.233
E74	44	M	PR	PTX+Capecitabine+αPD-1	24/8/2021	0.391
E76	68	F	PR	PTX+Cisplatin+αPD-1	26/8/2021	0.406
E85	66	M	PR	PTX+αPD-1	17/9/2021	0.278
E89	63	M	PR	PTX+Capecitabine+αPD-1	28/9/2021	0.471
E91	55	M	PR	TP+αPD-1	28/9/2021	0.232
E94	65	M	PR	PTX+Nedaplatin+αPD-1	12/10/2021	0.174
E100	76	M	PR	αPD-1	19/10/2021	0.405
E102	76	M	PR	αPD-1	21/10/2021	0.393
E104	66	M	PR	TP+αPD-1	26/10/2021	0.208
E106	55	M	PR	PTX+Cisplatin+αPD-1	28/10/2021	0.156
E107	76	F	PR	Tegafur+αPD-1	2/11/2021	0.185
E114	76	F	PR	mFOLFOX6+αPD-1	11/11/2021	0.573
E118	53	M	PR	PTX+Carboplatin+αPD-1	17/11/2021	0.309
E121	66	M	PR	PTX+Lobaplatin+αPD-1	22/11/2021	0.369
E124	61	M	PR	FOLFOX+αPD-1	2/12/2021	0.225
E129	39	M	PR	FOLFOX+αPD-1	10/12/2021	0.558
E135	69	M	PR	TP+αPD-1	11/1/2022	0.527
E136	67	M	PR	XELOXC1+αPD-1	12/1/2022	0.215

E139	57	M	PR	TP+αPD-1	18/1/2022	0.170
E140	69	M	PR	TP+αPD-1	8/2/2022	0.377
E144	58	M	PR	TP+αPD-1	22/2/2022	0.201
E145	70	M	PR	PTX+Capecitabine+αPD-1	23/2/2022	0.357
E147	73	M	PR	TP+αPD-1	28/2/2022	0.181
E156	66	M	PR	TP+αPD-1	14/3/2022	0.185
PD471	68	M	SD	PTX+Cisplatin+αPD-1	5/11/2020	0.226
PD314	66	M	SD	PTX+Capecitabine+αPD-1	17/9/2020	0.158
PD649	55	M	SD	PTX+Cisplatin+αPD-1	25/11/2020	0.246
PD747	54	M	SD	αPD-1	23/12/2020	0.130
PD287	63	M	SD	PTX+S-1+αPD-1	30/7/2020	1.069
PD1402	61	M	SD	αPD-1	1/5/2021	0.872
PD491	61	M	SD	αPD-1	18/11/2020	0.559
PD747	54	M	SD	αPD-1	23/12/2020	0.156
PD986	58	M	SD	αPD-1	3/2/2021	0.242
E12	60	M	SD	PTX+Cisplatin+αPD-1	18/3/2021	0.472
E14	44	M	SD	EGFR+PTX+Cisplatin	19/3/2021	0.195
E17	62	M	SD	PTX+Cisplatin+αPD-1	1/4/2021	0.160
E23	59	M	SD	αPD-1+TP	19/4/2021	0.216
E24	57	M	SD	PTX+Cisplatin+αPD-1	19/4/2021	0.269
E26	59	F	SD	αPD-1+EP	21/4/2021	0.493
E33	65	M	SD	PTX+Lobaplatin+αPD-1	7/5/2021	0.149
E46	54	M	SD	PTX+Cisplatin+αPD-1	3/6/2021	0.161
E57	67	M	SD	PTX+Nedaplatin+αPD-1	12/7/2021	0.300
E59	55	M	SD	PTX+Cisplatin+αPD-1	16/7/2021	0.166
E64	62	M	SD	PTX+Nedaplatin+αPD-1	23/7/2021	0.273
E86	58	M	SD	PTX+Cisplatin+αPD-1	22/9/2021	0.811
E93	59	M	SD	PTX+Nedaplatin+αPD-1	12/10/2021	0.425
E95	62	M	SD	PTX+Nedaplatin+αPD-1	13/10/2021	0.490
E96	59	M	SD	αPD-1+TP	14/10/2021	0.175
E109	62	M	SD	PTX+Capecitabine+αPD-1	9/11/2021	0.929
E110	56	F	SD	αPD-1+FOLFIRI	9/11/2021	0.317
E113	70	M	SD	PTX+Nedaplatin+αPD-1?	9/11/2021	0.160
E117	60	M	SD	αPD-1+TP	16/11/2021	0.255
E119	61	M	SD	PTX+Nedaplatin+αPD-1	18/11/2021	0.175
E132	64	M	SD	PTX+Capecitabine+αPD-1	21/12/2021	0.356
E150	69	M	SD	αPD-1+TP	3/3/2022	0.175
E151	69	M	SD	αPD-1+TP	3/3/2022	0.102

Table S2: ESCC patients details for PD-L1 and Fn detection

ID	Age	Male(M) or Female(F)	Curative Effect	Treatment	First treatment time	PD-L1 Positive Cells (%)	Δ*Ct*= *Ct*(Fn)-*Ct*(18S)
815517-13C	58	M	PD	Apatinib+αPD-1	9/8/2021	41.28% (Low)	5.34 (High)
809793-8E	62	F	PD	Docetaxel+Nedaplatin +αPD-1	13/11/2019	73.49% (High)	12.78 (Low)
775169	52	M	PD	Tinio+αPD-1	7/1/2020	68.25% (High)	8.85 (High)
601405	63	M	PD	Apatinib+αPD-1	19/5/2020	84.66% (High)	12.13 (Low)
844189	51	M	PD	Nedaplatin+Capecitabine +αPD-1	27/4/2021	41.05% (Low)	8.69 (High)
842008	68	M	PD	Lobaplatin+Paclitaxel +αPD-1	28/12/2021	33.11% (Low)	7.20 (High)
590103-1A	63	M	PD	Lobaplatin+Docetaxel +αPD-1	28/11/2019	48.64% (High)	8.74 (High)
736408-1E	63	M	PD	Nedaplatin+Paclitaxel +αPD-1	19/12/2020	46.56% (High)	0.51 (High)
714287-1F	55	M	PD	αPD-1	27/11/2019	40.96% (Low)	5.45 (High)
818457-8A	56	M	PD	αPD-1	7/1/2020	29.66% (Low)	14.20 (Low)
891440-5A	67	M	PR	Capecitabine+Paclitaxel +αPD-1	1/5/2021	31.9% (Low)	13.08 (Low)
821292-5B	62	M	PR	Capecitabine+Paclitaxel +αPD-1	26/12/2019	20.39% (Low)	7.28 (High)
892561-10C	56	F	PR	TP+αPD-1	18/6/2021	32.89% (Low)	15.21 (Low)
897011-13B	68	M	SD	Tinio+Paclitaxel+αPD-1	8/6/2021	41.95% (Low)	14.33 (Low)
890961-12F	57	M	SD	Capecitabine+Paclitaxel +αPD-1	29/4/2021	98.03% (High)	12.79 (Low)
822190-17B	68	M	SD	Paclitaxel+αPD-1	5/8/2020	40.20% (Low)	12.47 (Low)
804910-25B	68	M	SD	Nedaplatin+Paclitaxel +αPD-1	10/5/2021	47.52% (High)	12.75 (Low)
793334-2D	62	F	SD	Lobaplatin+Paclitaxel +αPD-1	22/4/2021	80.54% (High)	9.06 (High)
828118	64	M	SD	Tinio+Paclitaxel+αPD-1	24/3/2020	54.98% (High)	11.25 (Low)

Table S3: All antibodies used in flow cytometry analysis

Antibodies	Source	Identifier
CD274 (PD-L1, B7-H1) Monoclonal Antibody (MIH1), PE	eBioscience™, CA, USA	Cat# 12-5983-42<br>5 μl/test
Mouse IgG1 kappa Isotype Control (P3.6.2.8.1), PE	eBioscience™, CA, USA	Cat# 12-4714-82<br>5 μl/test
CD3 Monoclonal Antibody (17A2), FITC	eBioscience™, CA, USA	Cat#11-0032-82<br>0.25 μg/test
CD8a Monoclonal Antibody (53-6.7), PE	eBioscience™, CA, USA	Cat#12-0081-81<br>0.25 μg/test
APC Anti-Mouse CD4 Antibody [GK1.5]	Elabscience, Wuhan, China	Cat#E-AB-F1097E<br>5 μl/test
Granzyme B Monoclonal Antibody (NGZB), APC	eBioscience™, CA, USA	Cat#17-8898-80<br>0.125 μg/test
BV711 Mouse Anti-Human IFN-γ	BD Biosciences, NJ, USA	Cat#564039<br>5 μl/test
PE-Cy™7 Mouse Anti-Human TNF	BD Biosciences, NJ, USA	Cat#560923<br>5 μl/test

Table S4: All antibodies used in histology and immunofluorescence

Antibodies	Source	Identifier
Human anti-PD-L1	CST, MA, USA	Cat#13684<br>Dilution ratio:<br>1:400 (IF); 1:200 (IHC)
Mouse anti-PD-L1	GeneTex, SOCAL, USA	Cat#GTX31308<br>Dilution ratio:<br>1:1000 (IF); 1:400 (IHC)
Anti-ATF3	Abcam, UK	Cat#ab207434<br>Dilution ratio:<br>1:100 (IF); 1:100 (IHC)
α-Tubulin（MG17）Mouse Monoclonal Antibody	Ray antibody, Beijing, China	Cat#RM2007<br>Dilution ratio: 1:200
Rabbit anti-Fn*	Homemade	Dilution ratio: 1:1000
Mouse anti-Fn-Dps*	Homemade	Dilution ratio: 1:1000
DyLight 488 AffiniPure Goat Anti-Mouse IgG (H+L)	Fudebio, Hangzhou, China	Cat#FD0150<br>Dilution ratio: 1:200
DyLight 594 AffiniPure Goat Anti-Rabbit IgG (H+L)	Fudebio, Hangzhou, China	Cat#FD0129<br>Dilution ratio: 1:200
DyLight 488 AffiniPure Goat Anti-Rabbit IgG (H+L)	Fudebio, Hangzhou, China	Cat#FD0136<br>Dilution ratio: 1:200
Goat Anti-rabbit HRP	Fudebio, Hangzhou, China	Cat#FDR007<br>Dilution ratio: 1:200

*Validation of Fn and Fn-Dps antibodies specificity were shown in Figure S16.

Table S5: The sequences of primers for the qPCR analysis

Gene	Primer sequence	NCBI Reference Sequence
human *PD-L1*	F: 5'-AGAACTACCTCTGCACATCCTCCAA-3'<br>R: 5'-CCATTCCTTCCTCTTGTCACGCTCAG-3'	NM_001267706.2
mouse *Pd-l1*	F: 5'-TCACGGCTCCAAAGGACTTG-3'<br>R: 5'-CGTCTGTGATCTGAAGGGCA-3'	NM_021893.3
human *ATF3*	F: 5'- GGAGTGCCTGCAGAAAGAGT-3'<br>R: 5'- CCATTCTGAGCCCGGACAAT-3'	NM_001674.4
human *POU6F1*	F: 5'-AGATCCGGGAGTTTGCCAAG-3'<br>R: 5'-ATCAGGTTCTGCTGGCCTTC-3'	NM_001330422.2
human *CEBPB*	F: 5'-AAGCACAGCGACGAGTACAA-3'<br>R:5'-ACAGCTGCTCCACCTTCTTC-3'	NM_005194.4
human *NR4A2*	F:5'-GGACAACTACAGCACAGGCT-3'<br>R:5'-GCCACGTAGTTCTGGTGGAA-3'	NM_173173.3
human *MAFG*	F:5'-GGAGCTGGAGAAGCAGAAGG-3'<br>R:5'-GGGCATCCGTCTTGGACTTT-3'	NM_032711.4
human *GAPDH*	F:5'-GACTCATGACCACAGTCCATGC-3'<br>R:5'-AGAGGCAGGGATGATGTTCTG-3'	NM_001357943.2
Fn	F:5'-AAGCGCGTCTAGGTGGTTATGT-3;<br>R:5'-TGTAGTTCCGCTTACCTCTCCAG-3'	NR_117287.1
18S	F:5'-CAGCCACCCGAGATTGAGCA-3'<br>R:5'-TAGTAGCGACGGGCGGTGTG-3'	NC_000021.9

Table S6: All antibodies used in western blotting, Co-IP and ELISA

Antibodies	Source	Identifier
Human anti-PD-L1	CST, MA, USA	Cat#13684<br>Dilution ratio:<br>1:1000 (WB); 1:50 (IP)
Mouse anti-PD-L1	GeneTex, SOCAL, USA	Cat#GTX31308<br>Dilution ratio: 1:1000
Anti-ATF3	Abcam, UK	Cat#ab207434<br>Dilution ratio:<br>1:1000 (WB); 1:50 (IP)
GAPDH	Bioworld, MN, USA	Cat#AP0063<br>Dilution ratio: 1:5000
Lamin B1 (B-10)	Santa Cruz Biotechnology, CA, USA	Cat#sc-374015<br>Dilution ratio: 1:1000
α-Tubulin（MG17）Mouse Monoclonal Antibody	Ray antibody, Beijing, China	Cat#RM2007<br>Dilution ratio: 1:1000
Mouse anti-Fn-Dps*	Homemade	Dilution ratio: 1:1000
Goat Anti-rabbit HRP	Fudebio, Hangzhou, China	Cat#FDR007<br>Dilution ratio: 1:5000
IPKine™ HRP, Mouse Anti-Rabbit IgG LCS	Abbkine, CA, USA	Cat#A25022<br>Dilution ratio: 1:2000
IPKine™ HRP, Goat Anti-Mouse IgG HCS	Abbkine, CA, USA	Cat#A25112<br>Dilution ratio: 1:2000
IPKine™ HRP, Goat Anti-Rabbit IgG HCS	Abbkine, CA, USA	Cat#A25222<br>Dilution ratio: 1:2000
HRP, Goat Anti-Human IgG (H+L)	Earthox, CA, USA	Cat# E030170-01<br>Dilution ratio: 1:5000

* Validation of Fn-Dps antibody specificity was shown in Figure S16.

Table S7: Table summary of differentially expressed genes, related to Figure 6E

Gene	Famiy	Regulated	log2FC	*P* value
ATF3	TF_bZIP	up	1.68	2.76E-15
POU6F1	Pou	up	1.15	4.33E-03
CEBPB	TF_bZIP	up	0.98	1.89E-05
NR4A2	NGFIB-like	up	0.72	5.95E-03
MAFG	TF_bZIP	up	0.62	3.38E-03

FC, fold change
