# Supplementary material for: Intracellular Fusobacterium nucleatum infection attenuates antitumor immunity in esophageal squamous cell carcinoma
Source: Nat Commun. 2023 Sep 18;14:5788. doi: 10.1038/s41467-023-40987-3 (PMC10507087; doi:10.1038/s41467-023-40987-3)

Supplemental full uncut gels

PageRuler Prestained Protein Ladder (Thermo Scientific™, 26616)

Fig.1E

Antibodies used:

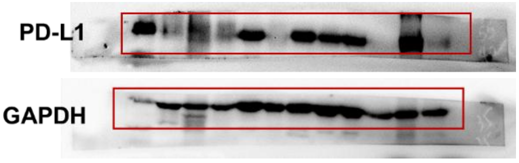

PD-L1 (Cell Signaling Technology, 13684); GAPDH (Bioworld, AP0063)

Fig.5I

Antibodies used:

PD-L1 (Cell Signaling Technology, 13684) for E109 and Kyse150;

GAPDH (Bioworld, AP0063); PD-L1 (GeneTex, GTX31308) for AKR

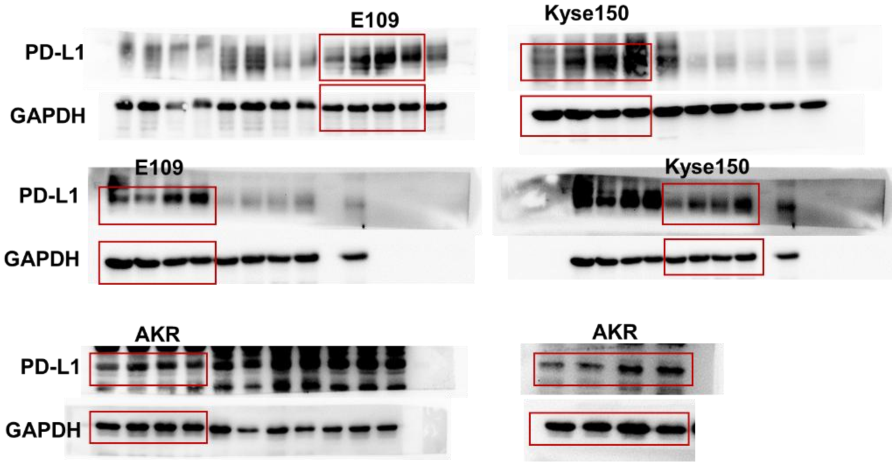

Fig.5K

Antibodies used:

PD-L1 (GeneTex, GTX31308); GAPDH (Bioworld, AP0063)

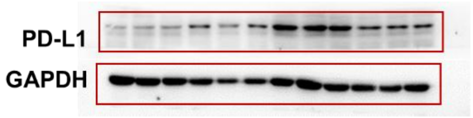

Fig.6B

Antibodies used:

PD-L1 (Cell Signaling Technology, 13684) for E109 and Kyse150;

GAPDH (Bioworld, AP0063); PD-L1 (GeneTex, GTX31308) for AKR

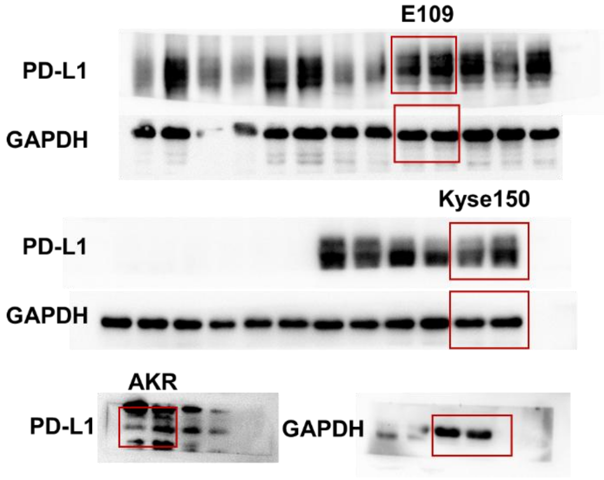

Fig.6H

Antibodies used:

ATF3 (Abcam, ab207434); PD-L1 (Cell Signaling Technology, 13684);

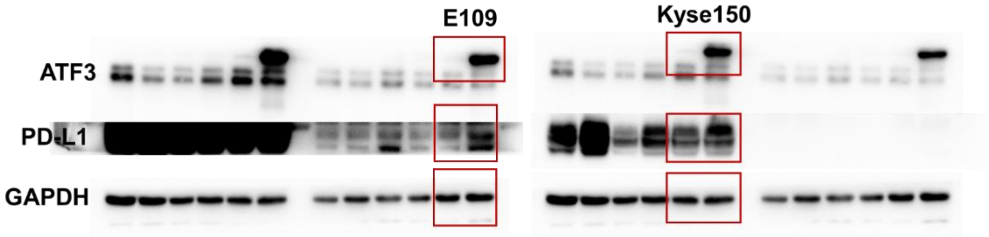

GAPDH (Bioworld, AP0063)

Fig.6I

Antibodies used:

ATF3 (Abcam, ab207434); PD-L1 (Cell Signaling Technology, 13684)

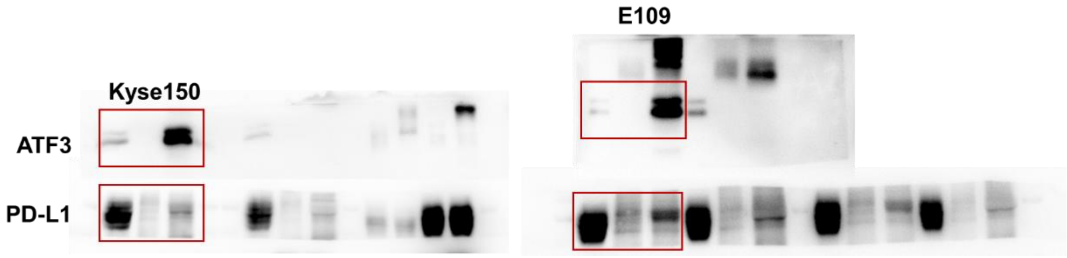

Fig.6J

Antibodies used:

ATF3 (Abcam, ab207434); PD-L1 (Cell Signaling Technology, 13684);

GAPDH (Bioworld, AP0063)

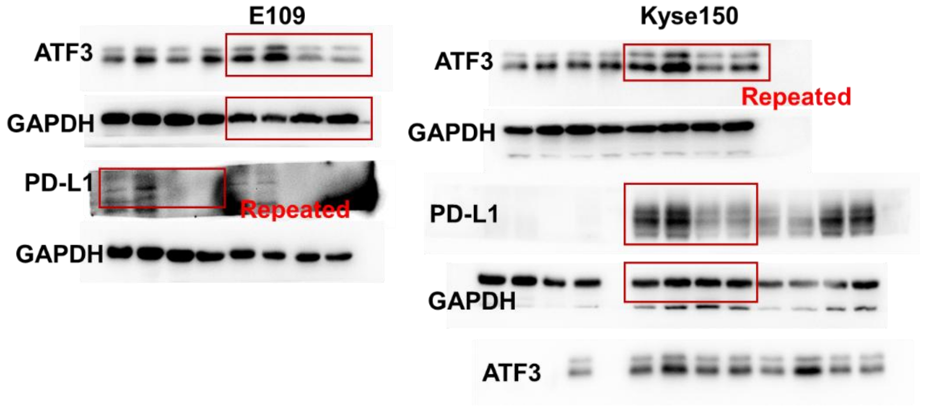

Fig.6M

Antibodies used:

Fn-Dps (Homemade); ATF3 (Abcam, ab207434)

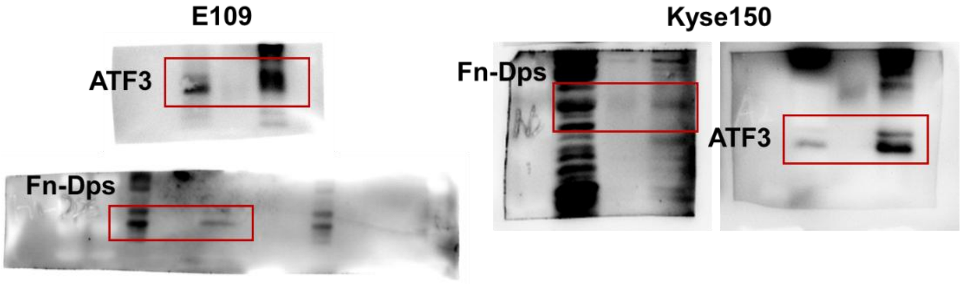

Fig.6Q

Antibodies used:

Fn-Dps (Homemade); Lamin B (Santa Cruz Biotechnology, sc-374015);

α-Tubulin (Ray antibody, RM2007)

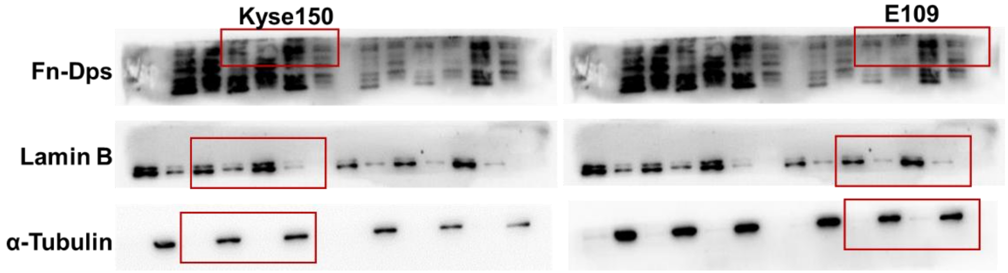

Fig.S10B

Antibodies used:

PD-L1 (Cell Signaling Technology, 13684); GAPDH (Bioworld, AP0063)

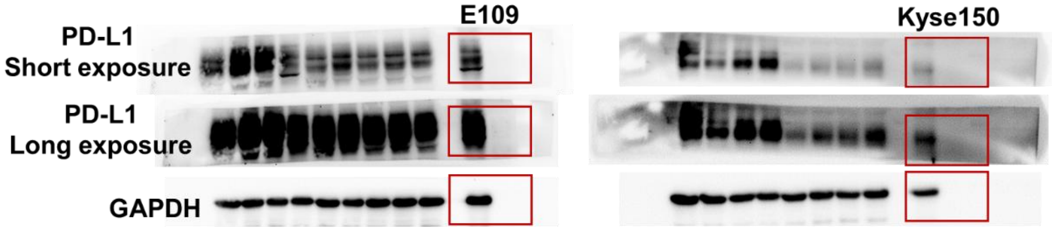

Fig.S10D

Antibodies used:

PD-L1 (Cell Signaling Technology, 13684); GAPDH (Bioworld, AP0063)

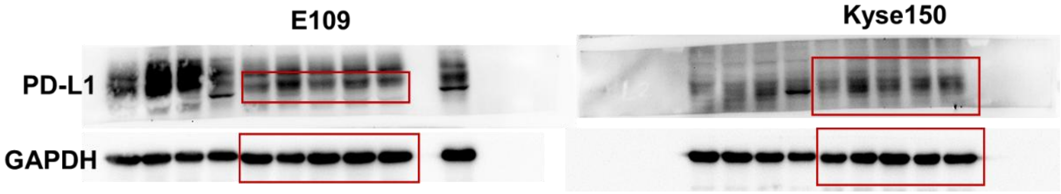

Fig.S12D

Antibodies used:

ATF3 (Abcam, ab207434); GAPDH (Bioworld, AP0063)

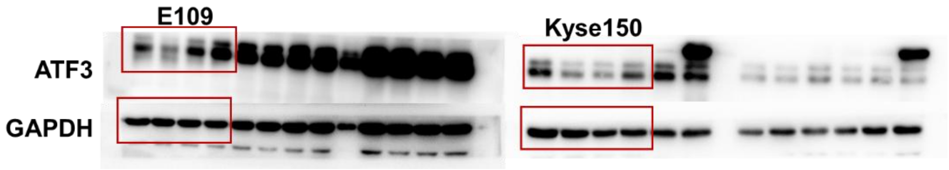

Fig.S14

Antibodies used:

Fn-Dps (Homemade); Fn (Homemade)

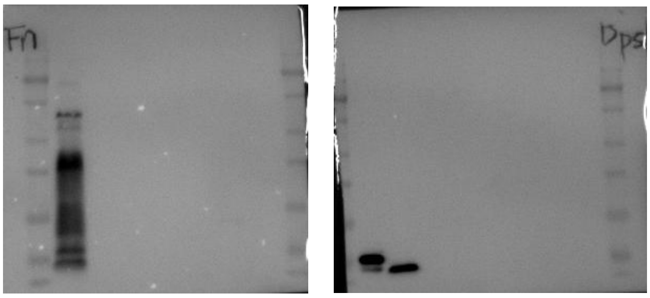

Supplement: Supplementary file 4 — Source Data [file 41467_2023_40987_MOESM4_ESM.zip › Source Data and full uncut gels/Supplemental full uncut gels.pdf]
